# Sustainable Optimization of Lightweight Aggregate Production from Bauxite Residue via Nonlinear Programming

**DOI:** 10.1021/acsomega.5c09834

**Published:** 2025-12-01

**Authors:** Larissa Pereira de Siqueira, Hugo Fernando Meiguins da Silva, Mailson Batista de Vilhena, Agenor Sousa Santos Neto, Bruno Marques Viegas, José Antônio da Silva Souza, Emanuel Negrão Macêdo

**Affiliations:** † Faculty of Chemical Engineering, 37871 Federal University of Pará, Belém 66075-110, Brazil; ‡ Department of Production Engineering, State University of Amapá, Macapá 68900-070, Brazil; § Graduate Program in Biotechnology, Federal University of Pará, Belém 66075-110, Brazil; ∥ Graduate Program in Process Engineering, Federal University of Pará, Belém 66075-110, Brazil

## Abstract

In this work, lightweight aggregates were developed from mixtures of bauxite residue and clay, using a nonlinear programming model to determine the optimum composition. The aggregates were characterized using TGA/DTG/DSC analysis, X-ray diffraction, scanning electron microscopy and physical tests of apparent density, porosity and water absorption, carried out on three samples (BR50, BR30.50 and BR0). The results showed that the bauxite residue favored the formation of amorphous and porous phases, reducing the density of the aggregates. A nonlinear programming model was developed to determine the optimal composition of a lightweight aggregate, resulting in a mixture containing 30.50% bauxite residue and 69.50% clay. This composition yielded a material with a density of 0.78 ± 0.03 g/cm^3^, porosity of 14.54 ± 1.13%, and water absorption of 14.05 ± 1.72%, confirming its classification as a lightweight aggregate. The reference sample, with 100% clay (BR0), had a density of 2.20 ± 0.02 g/cm^3^ and a more compact structure. The proposed model proved effective in maximizing density and porosity without compromising the integrity of the aggregate, indicating that the use of bauxite residue is a promising and sustainable alternative to produce materials for use in civil construction.

## Graphical abstract

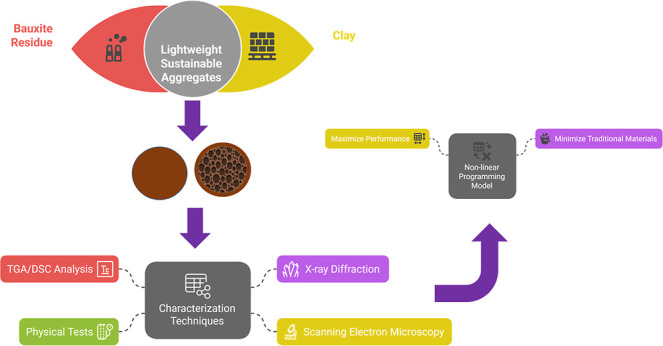

## 1 Introduction

Research indicates that industrial waste can be used as a raw material for sustainable development projects, reducing the use of nonrenewable natural resources.^1,2^ However, even with scientific advances, much of this waste is still underutilized due to the complexity of its composition and the lack of appropriate infrastructure for its management.^3^ Bauxite residue (BR), a byproduct of alumina refineries employing the Bayer process,^4,5^ exemplifies the challenges and opportunities associated with industrial waste management.

In addition to the costs and logistical difficulties associated with its large-scale disposal, regulatory constraints represent another factor responsible for the low reuse rate of bauxite residue, as they hinder its incorporation into production chains. Disposing of and storing this material on a large scale can represent around 5% of the total cost of alumina production.^6^ It is estimated that there is currently a global accumulation of approximately 3 billion tons of BR, to which an additional 120 million tons are added every year.^7,8^ In this context, the use of strategies that combine technological innovations and good management practices is essential to strengthen the competitiveness and sustainability of the industrial sector.^9^

Due to the high content of iron, sodium, aluminum, silicon, and titanium oxides in its composition,^10–12^ BR is a potential raw material for the production of lightweight aggregates. Each compound plays a significant role, particularly during the sintering process. Goethite, for example, decomposes into hematite, which, in combination with other fluxing agents, facilitates the reduction of the temperature for liquid-phase formation. Once this phase reaches adequate viscosity, it promotes the entrapment of the released gases and, consequently, the expansion of the aggregate. These aggregates could replace part of the natural aggregates used in the production of concrete, whose worldwide demand already reaches around 28 billion tons per year and could double in the next decade.^13^ Thus, converting BR into lightweight aggregates is part of a circular economy, since it reuses an industrial waste that is little exploited, reduces the demand for limestone, granite and basalt (rocks that are widely extracted from quarries for the production of natural aggregates) and also makes it possible to manufacture concrete with a lower mass, which is easier to transport and has superior thermal and structural performance.

Different types of mineral waste have already been used to make these aggregates, such as sediment from reservoirs, fly ash and limestone powder combined with fly ash.^14,15^ Interest is growing as researchers look for sustainable, high-performance solutions. Among the alternative raw materials studied are palm oil shells,^16^ sewage sludge,^17^ granite waste,^18^ fly ash,^19^ and granulated blast furnace slag.^20^ Lightweight aggregates meet the demand for materials with less mass and better thermal, hygrometric and acoustic insulation, favoring the development of lightweight mortars and external insulation systems.^21^

For the aggregates to have the right properties, it is essential that during sintering the silica (SiO_2_) and alumina (Al_2_O_3_) combine to form a glassy phase. This vitreous layer envelops the particles, enhancing surface resistance and reducing permeability. At the same time, certain fluxing agents (Fe_2_O_3_, Na_2_O, K_2_O, CaO and MgO) reduce the sintering temperature, which facilitates the softening and controlled expansion of the material. For these benefits to occur safely, the mass proportions between silica, alumina and fluxing agents need to be within well-defined ranges.^9,22^

Problems involving nonlinear programming have been widely explored in the literature across various processes, including separation processes,^23^ distribution networks,^24,25^ biomass polygeneration system,^26^ steam system,^27^ and electrical power systems.^28^ The main advantages of mathematical modeling for optimization include the reduction of operating costs, improvement in energy efficiency, rational use of resources, enhanced performance of production and logistics systems, and the ability to predict behavior under different scenarios and conditions. Moreover, nonlinear programming enables the incorporation of more realistic and complex relationships between variables, often nonlinear in nature, which are typical in industrial processes, energy systems, and environmental operations. As a result, mathematical optimization models have become essential tools for informed decision-making in contemporary projects and operational planning.

This paper integrates a nonlinear programming model with experimental validation to determine the maximum proportion of bauxite residue that can replace clay without compromising the chemical requirements for lightweight aggregate production. The high content of fluxing agents in bauxite residue promotes the formation of glassy phases, enhancing the efficiency of the sintering process.^29^ This approach enables the industrial production of more competitive lightweight aggregates, aligning waste management with economic and performance benefits for sustainable applications in civil construction.

## 2 Materials and Methods

### 2.1 Raw Materials and Sintering Process

To produce synthetic aggregates, bauxite residue and clay (originating from the Amazon region, it is widely employed in the manufacture of bricks for the construction industry) were dried in an oven at 105 °C for 24 h. Subsequently, each material underwent specific disaggregation: BR was milled in a ball mill for 30 min, while the clay was initially crushed and then refined using a disk mill. Both materials were classified by sieving through a 100 Tyler mesh (0.149 mm). Figure 1 shows the materials obtained after the pretreatment process.

**Figure 1:**
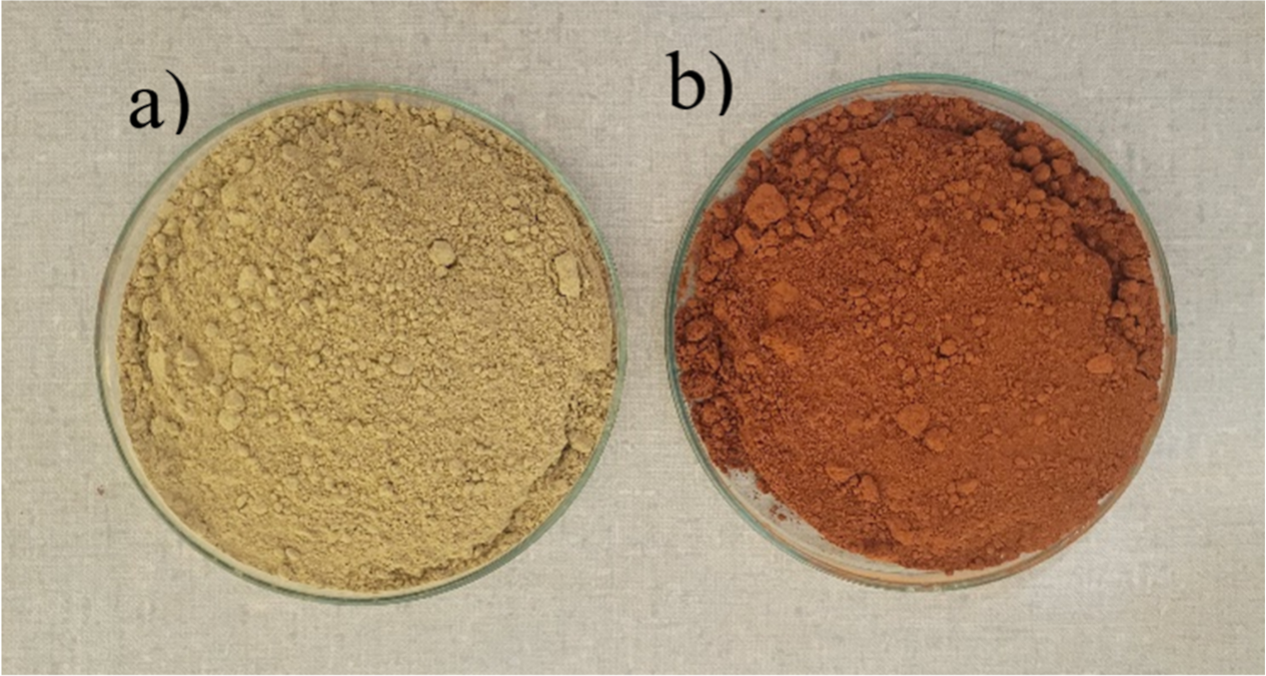
Raw materials processed to produce synthetic aggregates: (a) clay and (b) BR.

BR and clay were manually homogenized, followed by the gradual addition of water (≈20% of total mass that enabled the shaping of samples without exceeding moisture limits) to promote aggregate formation (produced manually). Based on preliminary tests and optimized ratios, two experimental formulations were defined, as summarized in Table 1. A third composition, derived from the optimization model, will be experimentally validated and discussed in the results section.

**Table 1:** Raw Material Compositions (wt %) Used in the Production of Synthetic Aggregates

sample	BR	clay
BR50	50	50
BR0	0	100

After formation, the aggregates were oven-dried for 24 h and sintered in an electric furnace (Jung, model JC4212). Heating was conducted at a rate of 10 °C/min up to 1250 °C, maintained for 2 h. Postsintering, the aggregates were characterized by thermal analysis (TGA/DSC), X-ray diffraction (XRD), scanning electron microscopy (SEM) and measurements of apparent density, porosity and water absorption. Figure 2 illustrates the flowchart detailing all stages of aggregate production and characterization.

**Figure 2:**
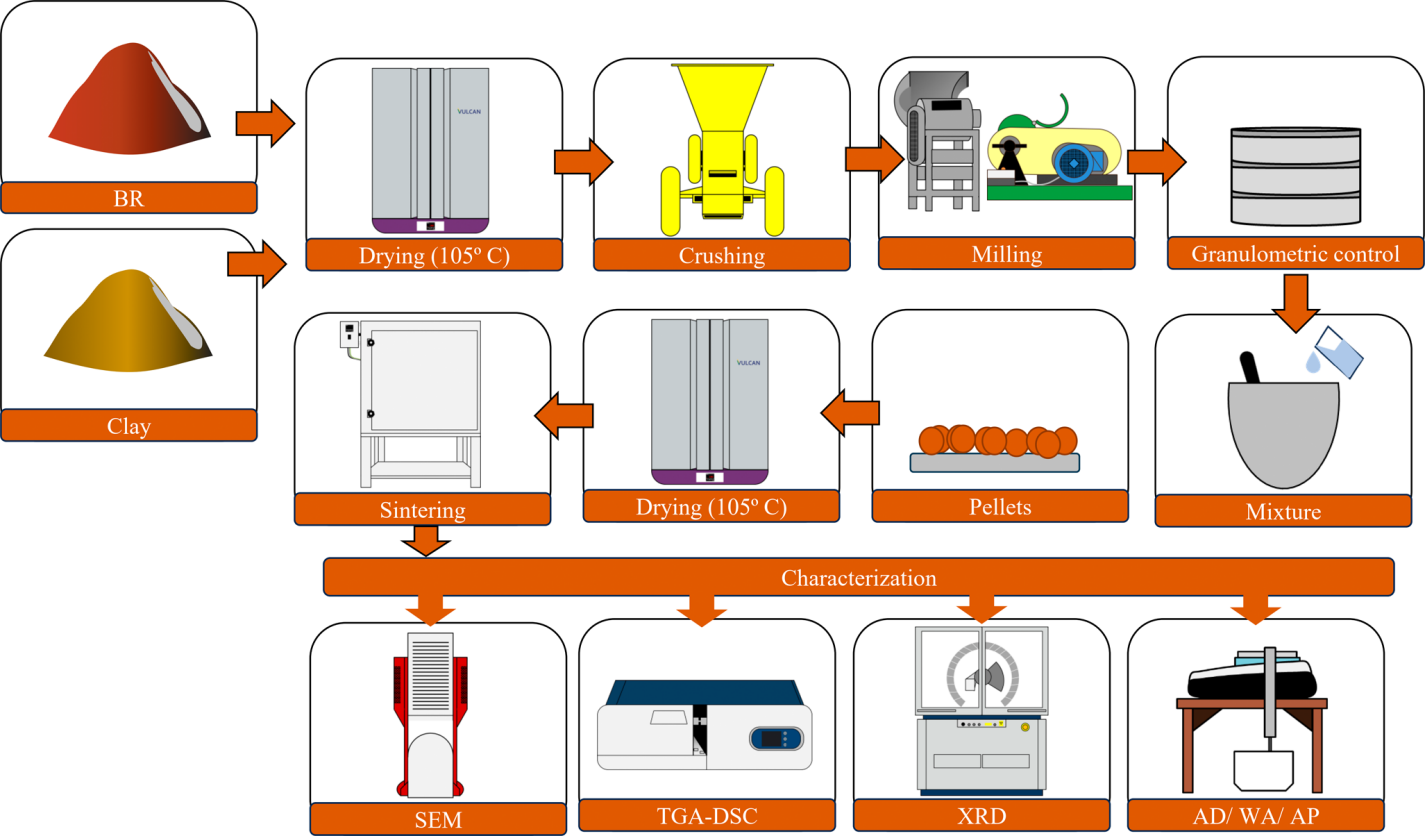
Flowchart of the production process and characterization of the synthetic aggregates.

### 2.2 Mathematical Model for Optimization

Based on studies of expanded clays,^9,13,15,30,31^ an optimization model was developed to maximize the bauxite residue content while complying with the constraints imposed on the SiO_2_, Al_2_O_3_, and ∑Flux contents. The composition limits were established on the basis of the X-ray fluorescence (XRF) results of the BR and clay obtained in previous work^32^ and are presented in Table 2. The decision variables were defined as the mass fractions of each raw material: BR *x*_BR_ and *x*_C_ for clay.

**Table 2:** Chemical Composition (wt %) of Major Oxides Identified in BR and Clay

oxide	BR	clay
Fe_2_O_3_	34.31	5.04
Al_2_O_3_	21.27	21.39
SiO_2_	17.72	63.57
Na_2_O	9.25	
TiO_2_	6.89	1.04
CaO	1.22	
MgO		0.527
LOI^a^	8.11	6.76

a Loss on igniton.

The model’s objective function aims to maximize the mass fraction of bauxite residue (*x*_BR_) in the ceramic mixture, while ensuring that the final composition remains within the ranges of SiO_2_, Al_2_O_3_ and ∑Flux considered ideal for the formation of light expandable aggregates. Therefore, in formal terms, the objective function can be defined according to eq 1

1 $$\max f\left(x\right)=x_{BR}$$

The model imposes a few restrictions to ensure its validity. The first establishes that the sum of the mass fractions of bauxite residue and clay corresponds to 100% of the mixture, as shown in eq 2

2 $$x_{BR}+x_{C}=1$$

According to the compositional limits established by Wang et al.^33^ and Tuan et al.^34^ for the expansive region of light clays (48–70% SiO_2_, 8–25% Al_2_O_3_, and 4.5 to 31% ∑Flux) the model imposes the following restrictions, which are illustrated in eqs 3–5:

3 $$a_{1}x_{BR}+a_{2}x_{C}\geq 0.48\;\text{and}\leq 0.7$$

4 $$a_{3}x_{BR}+a_{4}x_{C}\geq 0.08\;\text{and}\leq 0.25$$

5 $$a_{5}x_{BR}+a_{6}x_{C}\geq 0.045\;\text{and}\leq 0.31$$

where the coefficients *a*_1_ and *a*_2_ represent, respectively, the mass fractions of SiO_2_ in the BR and in the clay; *a*_3_ and *a*_4_ represent the Al_2_O_3_ contents; while *a*_5_ and *a*_6_ and refer to the ∑Flux content in each raw material. According to Fakhfakh et al.,^35^ the flux ratio (FR) is defined by the quotient between the overall SiO_2_ content and ∑Flux of the ceramic mixture as shown in eq 6

6 $$FR=\frac{C_{SiO_{2}}}{\sum Flux}$$

To ensure a glass phase viscosity capable of efficiently trapping the gases generated during sintering, the flow ratio must be greater than 2, according to Fakhfakh et al.^35^ In addition, the literature indicates that the ratio (Al_2_O_3_ + SiO_2_)/∑Flux should be between 3 and 10 to ensure adequate expansion of the aggregate.^30^ Thus, the following restriction presented in eq 7 is imposed

7 $$\frac{\left({Al}_{2}O_{3}+{SiO}_{2}\right)}{\left(\sum Flux\right)}\geq 3\;\text{and}\leq 10$$

This condition, incorporated into the optimization model, limits the composition of the mixture to a range of viscosity and reactivity favorable to the formation of light synthetic aggregates. Considering the theoretical relationships between the SiO_2_, Al_2_O_3_ and ∑Flux contents necessary for the formation of expansive light aggregates, the optimization problem is generally formalized in eq 8

8 $$\begin{array}{l} \left(\max\right)Z=x_{BR}\\ \{\begin{array}{l} x_{BR}+x_{C}=1\;\left(Total\;percentage\right)\\ a_{1}x_{BR}+a_{2}x_{C}\geq 0.48\;\text{and}\leq 0.7\;\left({SiO}_{2}\right)\\ a_{3}x_{BR}+a_{4}x_{C}\geq 0.08\;\text{and}\leq 0.25\;\left({Al}_{2}O_{3}\right)\\ a_{5}x_{BR}+a_{6}x_{C}\geq 0.045\;\text{and}\leq 0.31\;\left(\sum Flux\right)\\ {SiO}_{2}/\sum Flux>2\\ \frac{\left({Al}_{2}O_{3}+{SiO}_{2}\right)}{\sum Flux}\geq 3\;\text{and}\leq 10\\ x_{BR},x_{C}\geq 0 \end{array} \end{array}$$

According to the XRF results shown in Table 2, the SiO_2_ content is 17.72% in the BR (*a*_1_ = 0.1772) and 63.57% in the clay (*a*_2_ = 0.6357). Inserting these values into the restriction for silicon gives eq 9

9 $$0.1772x_{BR}+0.6357x_{C}\geq 0.48\;\text{and}\leq 0.7$$

By substituting the values obtained by XRF into the alumina inequality (*a*_3_ = 0.2127) and (*a*_4_ = 0.2139), the restriction is expressed by eq 10

10 $$0.2127x_{BR}+0.2139x_{C}\geq 0.08\;\text{and}\leq 0.25$$

In this study, the following melting oxides were considered: Fe_2_O_3_, Na_2_O, TiO_2_, CaO, and MgO. It is important to highlight that Fe_2_O_3_ is traditionally classified as a coloring oxide; however, some studies have reported its fluxing behavior^29,36,37^ under specific conditions. Similarly, it has been hypothesized that TiO_2_, when combined with other fluxing agents, may promote the formation of low-melting phases, thus acting secondarily as a flux. The individual contents of these oxides for bauxite residue and clay are shown in Table 2, generating the coefficients *a*_6_ (BR) and *a*_6_ (clay). Thus, the mass fraction of flux in the mixture is expressed by eq 11

11 $$\begin{array}{l} \sum Flux=[\left(0.3431x_{BR}+0.0504x_{C}\right)+\left(0.0925x_{BR}+0x_{C}\right)+\\ +\left(0.0689x_{BR}+0.01038x_{C}\right)+\left(0.0122x_{BR}+0x_{C}\right)+\\ +\left(0x_{BR}+0.00527x_{C}\right)] \end{array}$$

The flow mass fraction must be maintained within the range of 4.5 to 31% to ensure the desired expansion of the aggregate. Grouping *x*_BR_ and *x*_C_ and adding their coefficients gives eq 12

12 $$\sum Flux=0.5167x_{BR}+0.06605x_{C}\geq 0.045\;\text{and}\leq 0.31$$

Thus, by simultaneously imposing the ratios (Al_2_O_3_ + SiO_2_)/∑ Flux in the range 3–10 and SiO_2_/∑Flux > 2, the optimization problem is defined according to eq 13

13 $$\begin{array}{l} \left(\max\right)Z=x_{BR}\\ \{\begin{array}{l} x_{BR}+x_{C}=1\;\left(Total\;percentage\right)\\ 0.1772x_{BR}+0.6357x_{C}\geq 0.48\;\text{and}\leq 0.7\;\left({SiO}_{2}\right)\\ 0.2127x_{BR}+0.2139x_{C}\geq 0.08\;\text{and}\leq 0.25\;\left({Al}_{2}O_{3}\right)\\ 0.5167x_{BR}+0.06605x_{C}\geq 0.045\;\text{and}\leq 0.31\;\left(\sum Flux\right)\\ {SiO}_{2}/\sum Flux>2\\ \frac{\left({Al}_{2}O_{3}+{SiO}_{2}\right)}{\sum Flux}\geq 3\;\text{and}\leq 10\\ x_{BR},x_{C}\geq 0 \end{array} \end{array}$$

### 2.3 Thermal Analysis

The thermal characteristics of bauxite residue and clay were evaluated by thermogravimetry (TGA) and differential scanning calorimetry (DSC) on a Hitachi NEXTA STA300 simultaneous analyzer. The tests were conducted under an inert atmosphere (100 mL/min nitrogen flux rate), at 10 °C/min, in the range of 25 to 1250 °C.

### 2.4 X-Ray Diffraction

The mineralogical characterization of the raw materials and sintered aggregates was carried out by X-ray diffraction in an Empyrean diffractometer (PANalytical) operating at 40 kV and 35 mA, at room temperature. The aggregates were ground before the analysis to ensure adequate sample preparation. The data was collected with CuKα radiation (λ = 1.5418 Å) a step of 0.026° and a counting time of 30 s per step. X-ray diffraction graphs were then obtained using HighScore Plus software, which was used to identify the characteristic peaks of the phases present in the samples. To prepare and present the graphs, Origin software was used, where it was necessary to overlay the graphs with the data obtained from HighScore Plus, to facilitate visualization and identification of the corresponding peaks.

### 2.5 Scanning Electron Microscopy

The microstructure of the aggregates was analyzed using scanning electron microscopy at the scanning electron microscopy laboratory of the Museu Paraense Emílio Goeldi. Prior to imaging, the samples were sputter-coated with a thin layer of gold for 3 min. SEM images were acquired using a Tescan Mira 3 microscope equipped with a field emission gun (FEG), with an accelerating voltage of 15 kV and a working distance of approximately 15 mm.

### 2.6 Physical Properties (Density, Porosity and Water Absorption)

Ceramic properties were assessed according to ASTM C373-88:2006,^38^ following the procedures of Vilhena et al.^32^ and Brito et al.^39^ For each formulation, ten aggregates were selected and initially weighed in the dry state. The samples were then submerged in water for 24 h to determine the saturated mass. In contrast to previous studies that employed water, alcohol was used due to the low density of the aggregates (<1 g cm^–3^), which prevents accurate immersed weighing in water. Figure 3 illustrates the apparatus used to measure the immersed mass.

**Figure 3:**
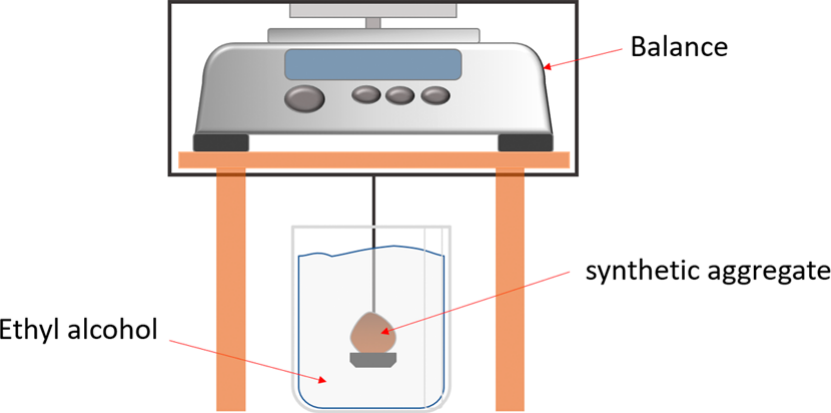
Synthetic aggregate submerged in ethyl alcohol for immersed mass measurement.

In this way, the apparent density (AD), water absorption (WA) and apparent porosity (AP) were calculated using eqs 14–16.

14 $$AD\left(g/cm^{3}\right)=\frac{M_{d}}{M_{W}-M_{S}}\rho_{ethyl\;alcohol}$$

15 $$WA\left(\%\right)=\frac{M_{W}-M_{d}}{M_{d}}100$$

16 $$AP\left(\%\right)=\frac{M_{W}-M_{d}}{M_{W}-M_{S}}100$$

where ρ_ethyl alcohol_ is the density of ethyl alcohol (0.88 g/cm^3^), *M*_d is_ the dry mass, *M*_w_ is the saturated mass and *M*_s_ is the immersed mass of the sample.

## 3 Results and Discussion

### 3.1 Optimization Results and Ternary Diagram

The nonlinear programming model defined in eq 13 was solved using the Microsoft Excel Solver with the GRG Nonlinear algorithm. This tool enables configuration of the objective function and constraints, as well as the selection of appropriate algorithms for nonlinear systems. The implementation converged to optimal mass fractions of *x*_BR_ = 30.50% and *x*_C_ = 69.50%, while satisfying the compositional limits for SiO_2_, Al_2_O_3_ and the melting oxides. Table 3 shows the final concentrations of the main oxides for the optimized formulation.

**Table 3:** Oxide Composition (wt %) of the Optimized Lightweight Aggregate Formulation (BR30.50)

Al_3_O_2_	SiO_2_	Flux	SiO_2_/Flux	(Al_2_O_3_+SiO_2_)/Flux
21.35	49.59	23.65	2.10	3.00

The optimized formulation contains 21.35% Al_2_O_3_ and 49.59% SiO_2_, indicating a high concentration of refractory components that confer thermal resistance and structural stability. The molten oxide content is 23.65%, which reduces the sintering temperature and favors the formation of glassy and porous phases. The SiO_2_/Flux ratio reached 2.10, while the (Al_2_O_3_ + SiO_2_)/Flux ratio was 3.00. This last parameter, known as the melting index, quantifies the balance between the network formers and the melting oxides. An adequate melt index adjusts the viscosity of the liquid phase during sintering, reducing the melting point and ensuring the controlled retention of evolved gases, which is fundamental for the expansion and formation of porous structures in the ceramic matrix.

Figure 4 shows the Riley ternary diagram,^40^ in which the vertices correspond to the concentrations of SiO_2_, Al_2_O_3_ and melting oxides. The dotted area delimits the ideal compositional range to produce lightweight expandable aggregates. Formulations whose chemical coordinates fall within this region favor the formation of a glassy phase and the controlled retention of gases during sintering, ensuring the desired expansion and porosity in the material.^39,40^

**Figure 4:**
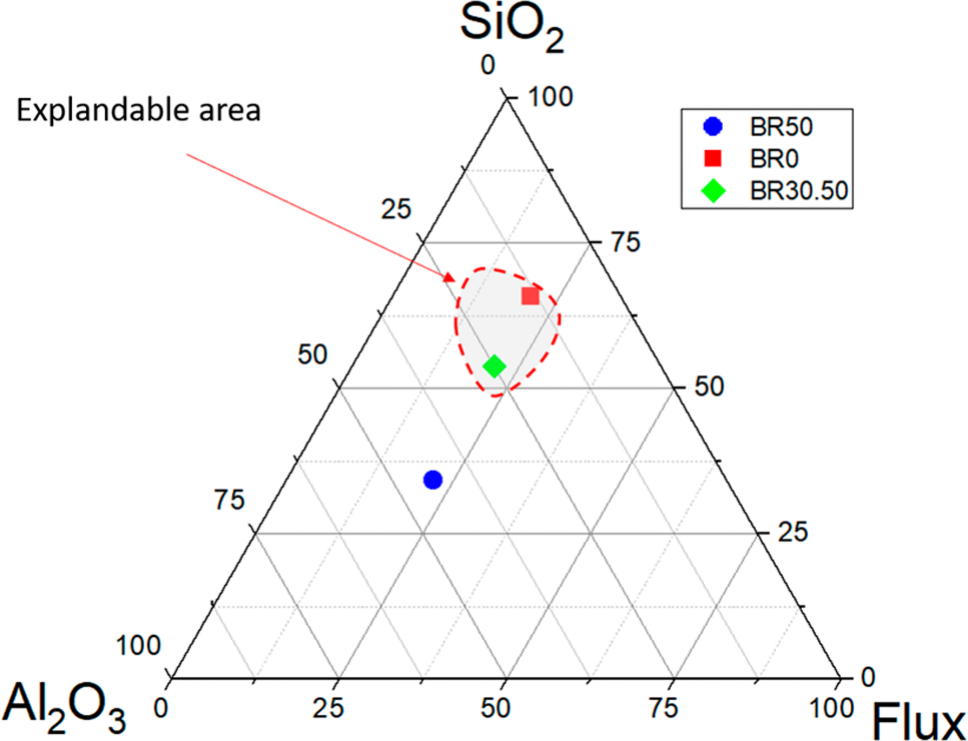
Riley ternary diagram with BR50, BR0, and BR30.50 formulations and the ideal expansion zone for lightweight aggregates.

As shown in Figure 4, the BR30.50 and BR0 formulations fall within the area favorable to the expansion of lightweight aggregates, while the BR50 sample remains outside this region. The optimization model indicated an optimal bauxite residue fraction of 30.50%, a percentage close to that identified by Chunguang Song et al.,^29^ who indicated 30% as the ideal proportion for expandable aggregates. However, despite BR0 being in the expandable range of the diagram, this formulation did not show expansion, which suggests that this phenomenon depends not only on the ternary composition, but also on the interaction between constituents, sintering kinetics and the presence of melting oxides that reduce the viscosity of the liquid phase and promote the generation of pores. The lack of expansion in BR0 can therefore be attributed to the low content of fluxing oxides in the clay used.

### 3.2 Thermal Behavior of Materials

Thermogravimetric and differential exploratory calorimetry analyses were carried out on BR and the clay. The resulting profiles are shown in Figure 5.

**Figure 5:**
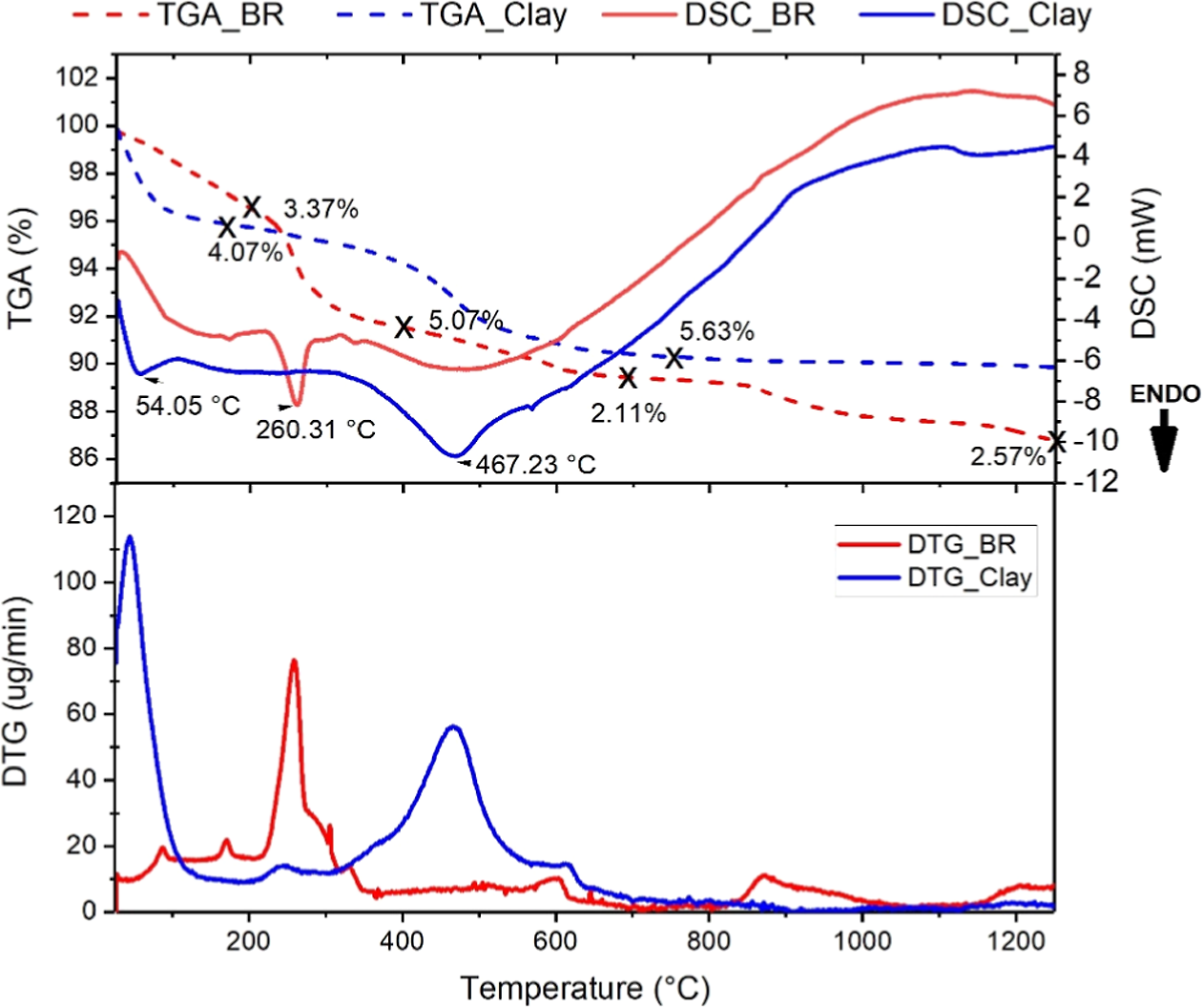
–TGA, DTG and DSC analysis of BR and clay.

TGA analysis of the BR revealed three distinct stages of mass loss during heating. The first stage occurs between 50 and 200 °C, with a mass loss of 3.37%, attributed to the removal of adsorbed water and the dehydroxylation of gibbsite.^41^ The second stage, between 200 and 700 °C, is associated with the transformation of goethite into hematite, resulting in an additional mass loss of 5.07%. The third stage, from 700 to 1200 °C, with a loss of 2.57%, the decomposition of volatile species retained within the sodalite structure occurs, a characteristic feature of bauxite residues.^41^

These processes are accompanied by thermal variations observed in the DSC curve. The first endothermic peak appears between 50 and 230 °C and is associated with the release of surface water. Next, a second peak appears around 260 °C, indicating the transformation of gibbsite into a transitional alumina phase (χ-Al_2_O_3_).^42^ Between 500 and 700 °C, thermal effects may be related to the decomposition of calcite and the transformation of α-quartz into β-quartz,^43^ while between 800 and 1100 °C, a thermal signal is observed that may indicate the formation of nepheline.^41^

For the clay sample, the TGA curve indicates an initial mass loss of 4.37% between 50 and 200 °C, related to the evaporation of physically adsorbed water. Between 200 and 700 °C, the main thermal transformation occurs, with a mass loss of 2.11%, attributed to the dehydroxylation of kaolinite and the formation of metakaolinite.^44^ This process is evidenced by an endothermic peak around 467 °C in both DTG and DSC curves. In the 700 to 1200 °C range, the clay shows a mass loss of 5.63%, indicating more complex structural transformations and the formation of new mineral phases. The DSC curve supports these findings, with a first endothermic peak below 100 °C, related to water loss, followed by a broad peak between 200 and 700 °C, corresponding to the dehydroxylation of kaolinite.

### 3.3 Mineralogical Analysis by XRD

The X-ray diffractograms of the bauxite residue (Figure 6a) and the clay (Figure 6b) are shown in Figure 6.

**Figure 6:**
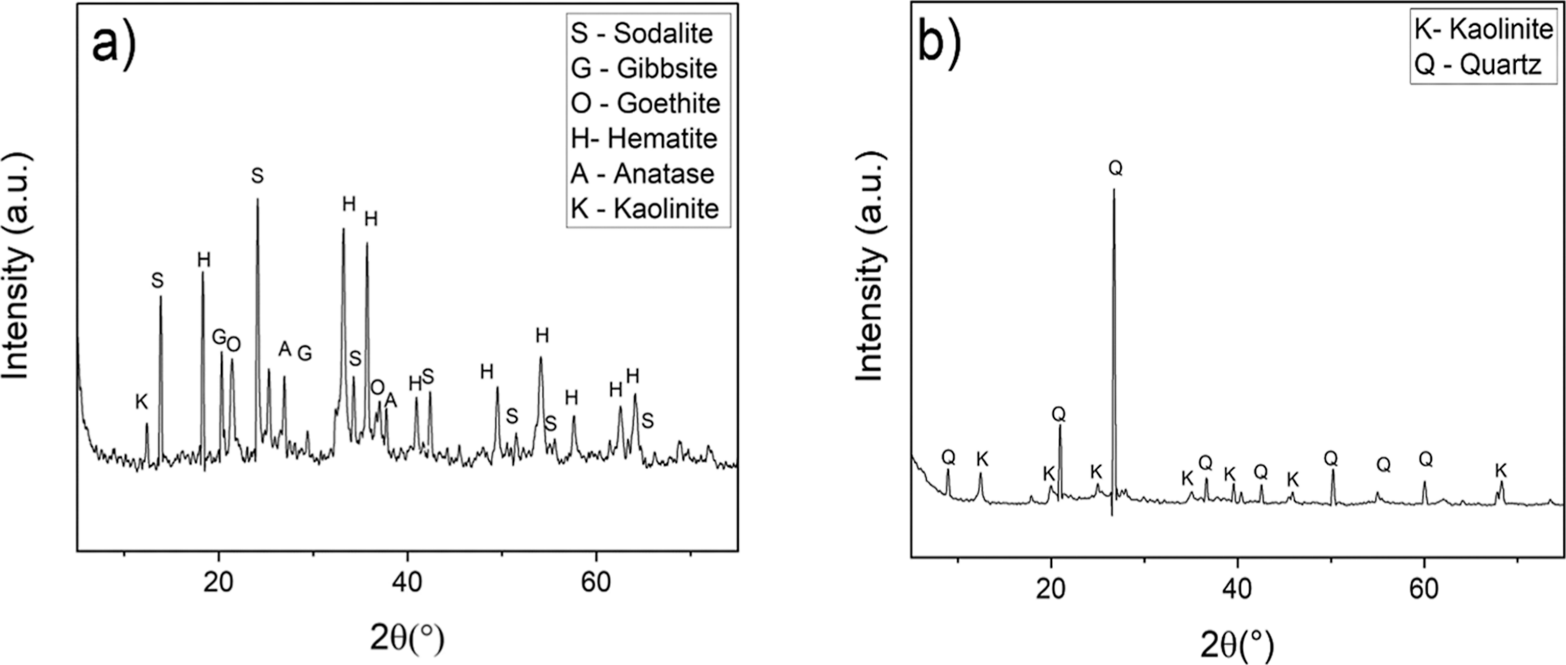
X-ray diffraction patterns of: (a) BR and (b) clay.

X-ray diffraction analysis of the bauxite residue (Figure 6a) revealed a diverse set of crystalline phases, including sodalite (Na_2_OAl_2_O_3_SiO_2_, PDF 01-082-1812), gibbsite (Al­(OH)_3_, PDF 00-007-0324), goethite (FeO­(OH), PDF 01-074-2195), hematite (Fe_2_O_3_, PDF 01-079-0007), anatase (TiO_2_, PDF 01-084-128600) and kaolinite (Al_2_Si_2_O_5_(OH)_4_, PDF 01-083-0971). These phases reflect the diverse mineralogical composition of the waste, which is characterized by aluminum and iron oxides and hydroxides, as well as secondary silicates that can directly influence the thermal behavior and reactivity during sintering.

XRD analysis of the clay (Figure 6b) revealed predominantly quartz (SiO_2_, PDF 01-086-1628) and kaolinite (Al_2_Si_2_O_5_(OH)_4_, PDF 00-029-1488), showing a high content of aluminum silicates typical of ceramic clays. The presence of kaolinite is important because it imparts plasticity to the material during processing and, upon heating, transforms into phases such as metakaolinite and mullite, which enhance thermal and mechanical resistance, thereby improving its applicability in ceramics and other industrial sectors. Figure 7 shows the diffractograms of the synthetic aggregates (BR50, BR30.50 and BR0), highlighting the crystalline phases formed after sintering.

**Figure 7:**
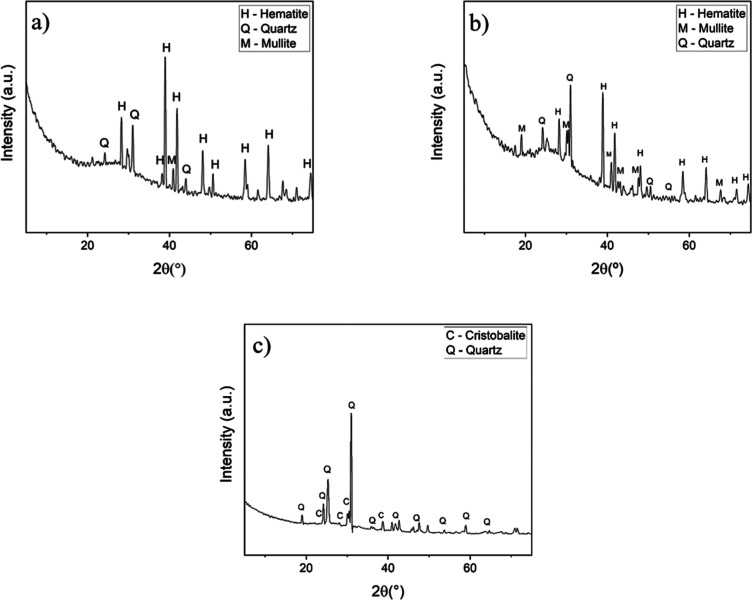
X-ray diffraction patterns of the samples: (a) BR50, (b) BR30.50, and (c) BR0.

X-ray diffraction analysis of the synthesized aggregates revealed the coexistence of multiple crystalline phases, influenced by the raw material composition and sintering conditions. Additionally, the increased diffuse background in specific regions indicates the presence of amorphous phases (see Supporting Information Figure S1). In the diffractogram of sample BR50 (Figure 7a), prominent peaks of hematite (Fe_2_O_3_, PDF 01-085-0599), mullite (Al_4_SiO_8_, PDF 01-073-1389), and quartz (SiO_2_, PDF 01-085-0335) were identified.

In sample BR30.50 (Figure 7b), the crystalline phases identified include hematite (Fe_2_O_3_, PDF 01-085-0599), quartz (SiO_2_, PDF 01-083-2465), and mullite (Al_4_SiO_8_, PDF 01-073-1389). A diffuse halo between 10° and 30° 2θ confirms a significant amorphous fraction. The presence of mullite indicates a reaction between clay components (mainly from the kaolinite phase) that enhances nucleation, whereas the amorphous phase points to partial vitrification of uncrystallized constituents, producing a heterogeneous ceramic matrix. The clay-mineral fraction imparts plasticity and yields both mullite and a glassy phase during sintering.^45^

In the sample composed exclusively of clay (Figure 7c), crystalline peaks of quartz (SiO_2_, PDF 01-083-2465) and cristobalite (SiO_2_, PDF 01-076-0941) stand out, with a reduction in diffuse background scattering, indicating a minimal amorphous fraction. The formation of cristobalite indicates that the quartz has been transformed, resulting in a predominantly crystalline microstructure.

### 3.4 Morphological Characterization by SEM

Figure 8 presents the scanning electron microscopy micrographs of the synthetic aggregates, highlighting their morphological features and pore distribution.

**Figure 8:**
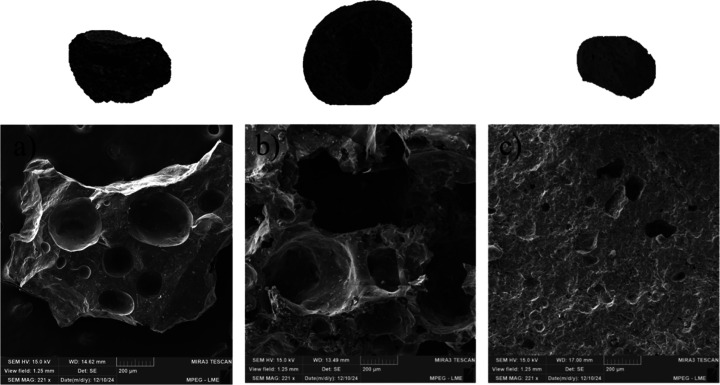
Microstructure of the aggregates: (a) BR50, (b) BR30.50, and (c) BR0.

The SEM micrograph of the BR50 sample (Figure 8a) shows a network of interconnected macropores, characterized by spherical voids and surface cavities throughout the granule structure. This high porosity results from the thermal decomposition of bauxite constituent phases and the release of gases, mainly volatiles during sintering, which promote the formation of micrometer-sized pores. The presence of Fe_2_O_3_ in the BR can react with SiO_2_ and Al_2_O_3_ to generate viscous phases that retain the gases and, consequently, promote aggregate expansion.^46^

The SEM micrograph of sample BR30.50 (Figure 8b) shows a more heterogeneous porosity pattern, interspersed with zones of partial fusion of the clay phase. This morphology suggests that the lower residue content enhances sintering efficiency, reducing average pore diameter and interconnectivity. Compared to sample BR50, the limited incorporation of bauxite residue in BR30.50 restrains macropore formation, resulting in a ceramic matrix with finer porosity and improved structural cohesion.

The SEM micrograph of the pure clay sample (Figure 8c) reveals an almost complete absence of pores, with a smooth and highly compact surface. This behavior indicates that, in the absence of bauxite residue, sintering leads to the full fusion of clay particles, producing a densified ceramic body with no pore formation typically associated with gas release or decomposition of residue components.

The results indicate that the microstructure of the aggregates is strongly influenced by the proportion between bauxite residue and clay. A higher content of bauxite residue promotes macropore formation and reduces apparent density, while an increased amount of clay enhances particle coalescence, resulting in a denser matrix. The production of lightweight aggregates from clay depends on the temperature range in which pyroplastic deformation, gas generation, and gas retention occur simultaneously. These gases originate from the thermal decomposition of carbonates, the reduction of ferric oxides, the combustion of organic matter, and the release of structural water. Swelling results from the retention of these gases within a viscous silicate phase, influenced by the flux content, which controls pyroplasticity, and by the SiO_2_/Σflux ratio, which affects both viscosity and the formation of a vitrified shell capable of retaining the gases.^47^ Therefore, adjusting the relative amounts of bauxite residue and clay enables systematic control of porosity, allowing the final properties of the aggregates to be tailored to specific performance requirements.

### 3.5 Evaluation of Physical Properties

The physical properties of the samples were evaluated by determining their apparent density, apparent porosity, and water absorption. The results are presented in Table 4.

**Table 4:** Average Values of Apparent Density, Apparent Porosity, and Water Absorption for the Aggregate Samples

sample	AD (g/cm^3^)	AP (%)	WA (%)
BR50	1.21 ± 0.06	11.48 ± 2.79	9.29 ± 2.70
BR30.50	0.78 ± 0.03	14.54 ± 1.13	14.04 ± 1.72
BR0	2.20 ± 0.02	6.76 ± 1.22	3.08 ± 0.59

As shown in Table 4, the sample composed exclusively of clay exhibited the highest apparent density (2.20 ± 0.02 g/cm^3^), indicating a high degree of compactness. In contrast, the optimized formulation with 30.50% bauxite residue showed the lowest value (0.78 ± 0.03 g/cm^3^), reflecting low density and high porosity. The sample with 50% BR exhibited an intermediate density of 1.21 ± 0.06 g/cm^3^, confirming that increasing the bauxite residue content reduces the apparent density of the ceramic matrix up to a critical (optimum) point, after which it begins to rise again. Incorporating bauxite residue increased the samples’ apparent porosity to 6.76 ± 1.22% (BR0), 11.48 ± 2.79% (BR50), and 14.54 ± 1.13% (BR30.50). This higher porosity results from the residue’s morphology and its thermal reactivity during sintering, which generate gas and promote the controlled formation of internal pores. The thermal decomposition of carbonates releases carbon dioxide (CO_2_), whereas the reduction or dissociation of ferric oxides generates oxygen and other gaseous byproducts. The combustion of organic matter contributes gases such as CO_2_ and CO, and the release of interlayer water molecules occurs at different temperature ranges. The combination of these reactions, when occurring simultaneously with the pyroplastic phase of the matrix, promotes gas retention and the consequent development of porosity.

The BR30.50 formulation exhibited the highest absorption value (14.04 ± 1.72%), followed by the BR50 (9.29 ± 2.70%), while the pure clay sample (BR0) showed the lowest water absorption (3.08 ± 0.59%). These results confirm the direct relationship between porosity and water retention, demonstrating that the increase in internal cavities promotes greater water uptake.

As shown in Figure 9, the apparent density decreased progressively as the clay was replaced by bauxite residue, reaching a minimum level beyond which no significant reductions were observed. This behavior is directly associated with the greater intrinsic porosity of BR and the formation of voids during sintering. The reduction in density follows the expected trend for ceramic systems incorporating porous mineral waste. Additional measurements conducted on alternative BR proportions confirmed this trend, reinforcing the understanding of how composition variations influence the material’s density.

**Figure 9:**
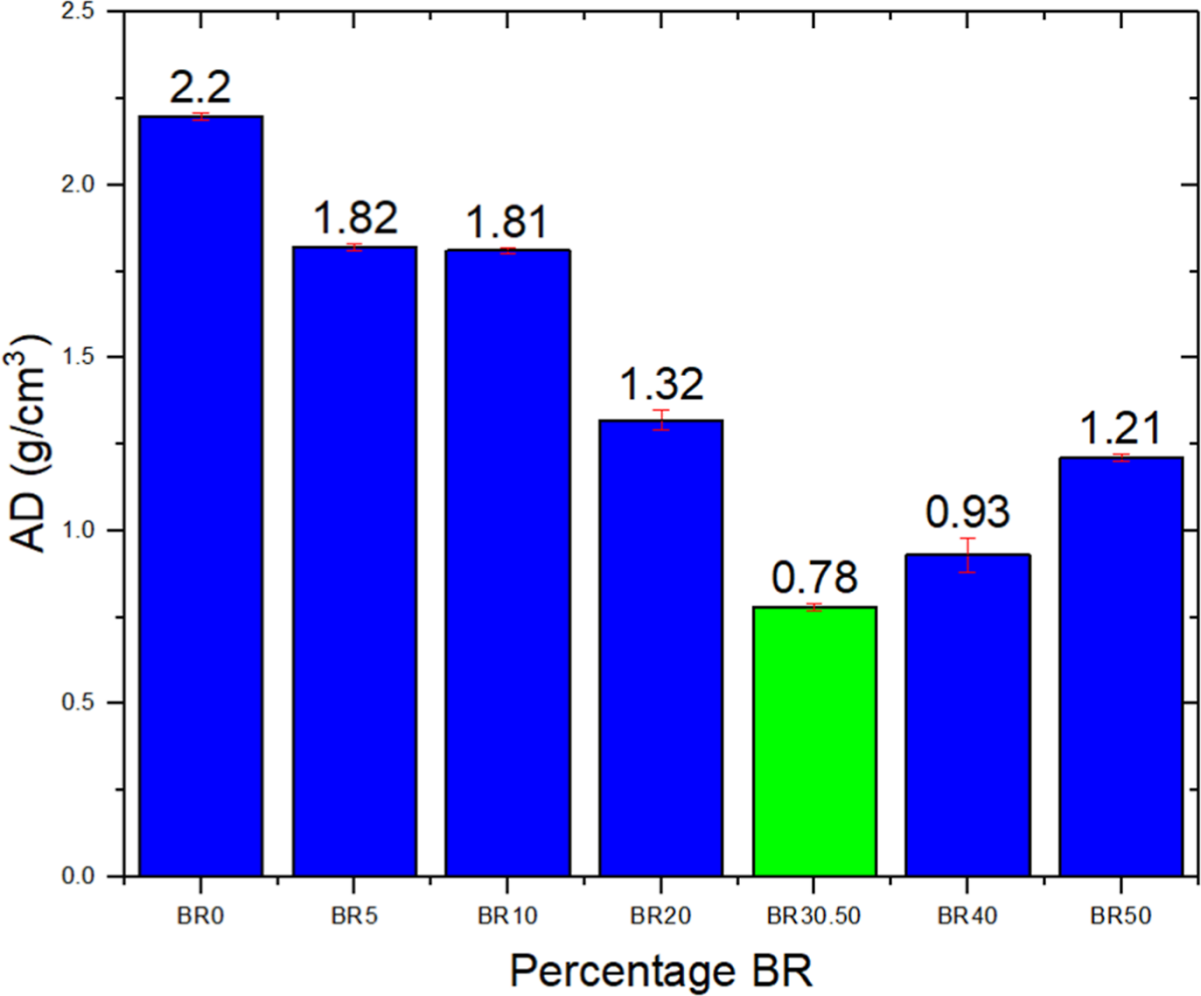
Apparent density measurements of aggregates with varying BR concentrations.

The optimization model results aligned with the experimental data presented in Figure 9, confirming that the partial substitution of clay with bauxite residue significantly affects material density. A marked reduction in density was observed up to a critical point (most evident in the BR30.50 composition), followed by a small increase at higher BR contents. This behavior suggests the existence of an optimum substitution point, in which the incorporation of BR minimizes density without excessively compromising structural integrity.

This behavior is consistent with the study conducted by Jiannan Pei et al.,^46^ who observed that the swelling index initially increases and then decreases as the bauxite residue content increases, reaching a maximum value when the BR content is 40%. However, the viscosity of the liquid phase decreases with the increase in Fe_2_O_3_ content when the BR content exceeds 40%, and the lower viscosity of the liquid phase cannot retain gas effectively, which reduces the aggregate’s expansion. Thus, the higher the BR content, the lower the viscosity and swelling index. This result is relevant for applications requiring lightweight materials, such as thermal insulation components or lightweight structures.

## 4 Conclusion

This study demonstrated the feasibility of using bauxite residue as a raw material for producing lightweight aggregates through its combination with clay and subsequent sintering. XRD analysis confirmed the formation of crystalline phases (hematite, quartz, kaolinite, anatase and mullite), as well as diffuse diffraction regions indicative of amorphous fractions generated by thermal transformations. SEM micrographs corroborated that increasing the BR content enhanced macroporosity, while the pure clay sample exhibited a dense and compact microstructure.

The physical properties varied with composition. The pure clay sample exhibited higher apparent density (2.20 ± 0.02 g/cm^3^), lower porosity (6.76 ± 1.22%), and low water absorption (3.08 ± 0.59%), typical of dense ceramic bodies. The equimolar mixture (BR50) presented intermediate values (1.21 ± 0.06 g/cm^3^; 11.48 ± 2.79%; 9.29 ± 2.70%), while the optimized formulation (BR30.50) showed the lowest density (0.78 ± 0.03 g/cm^3^), highest porosity (14.54 ± 1.13%), and maximum water absorption (14.04 ± 1.72%), confirming its suitability as a lightweight aggregate.

The integration of the nonlinear programming model with experimental data proved effective in identifying the optimal composition, enabling the selection of proportions that simultaneously maximize porosity and minimize apparent density, key requirements for lightweight aggregates. By valorizing an industrial byproduct, the proposed methodology aligns with sustainability and circular economy principles, supporting the development of high-performance ceramic materials for civil construction.

## Supplementary Material



## Notes

Figure S1 presents the XRD patterns of the samples before and after the sintering process at 1250 °C (PDF)

## Nomenclature

Al­(OH)_3_: Gibbsite
Al_2_O_3_: alumina
Al_2_Si_2_O_5_(OH)_4_: kaolinite
AP: apparent porosity
ASTM: american society for testing and materials
AD: apparent density
BR: bauxite residue
C: clay
CaO: calcium oxide
DTG: derivative thermogravimetry
DSC: differential scanning calorimetry
Fe_2_O_3_: hematite
FeO­(OH): goethite
FR: flow ratio
GRD: generalized reduced gradient
LOI: loss on ignition
*M*_d_: dry mass
MgO: magnesium oxide
*M*_s_: immersed mass
*M*_w_: saturated mass
Na_2_O: sodium oxide
Na_2_O·Al_2_O_3_·SiO_2_: sodalite
SEM: scanning electron microscopy
SiO_2_: silicon dioxide
TGA: temogravimetry analysis
TiO_2_: anatase
WA: water absorption
*x*_BR_: bauxite residue concentration
*x*_C_: clay concentration
XRD: X-ray diffraction
