# Supplementary material for: Sustainable Optimization of Lightweight Aggregate Production from Bauxite Residue via Nonlinear Programming
Source: ACS Omega. 2025 Dec 1;10(48):59829–39. doi: 10.1021/acsomega.5c09834 (PMC12771972; doi:10.1021/acsomega.5c09834)
Supplement: Supplementary file 1 [file ao5c09834_si_001.pdf]

Supplementary material for: Sustainable optimization of lightweight aggregate production from bauxite residue via nonlinear programming

Larissa Pereira de Siqueira[1], Hugo Fernando Meiguins da Silva[1], Mailson Batista de Vilhena[2], Agenor Sousa Santos Neto[2], Bruno Marques Viegas[3,4], José Antônio da Silva Souza[4], Emanuel Negrão Macêdo[1,4,*]

[1] Faculty of Chemical Engineering, Federal University of Pará, Belém, PA, 66075-110, Brazil.

[2] Department of Production Engineering, State University of Amapá, Macapá, AP 68900-070, Brazil

[3] Graduate Program in Biotechnology, Federal University of Pará, Belém, PA, 66075-110, Brazil.

[4] Graduate Program in Process Engineering, Federal University of Pará, Belém, PA 66075-110, Brazil.

Email addresses

larissa.siqueira@itec.ufpa.br (Larissa Pereira de Siqueira), hugomeiguins220@gmail.com (Hugo Fernando Meiguins da Silva), mailson.vilhena@ueap.edu.br (Mailson Batista de Vilhena), agenor.neto@ueap.edu.br (Agenor Sousa Santos Neto), viegasmbruno@gmail.com (Bruno Marques Viegas), jass@ufpa.br (José Antônio da Silva Souza), enegrao@ufpa.br (Emanuel Negrão Macêdo).

* Corresponding author.

Mailing address: Faculty of Chemical Engineering, Federal University of Pará, Belém, PA, 66075-110, Brazil.

Email address: enegrao@ufpa.br (Emanuel Negrão Macêdo).

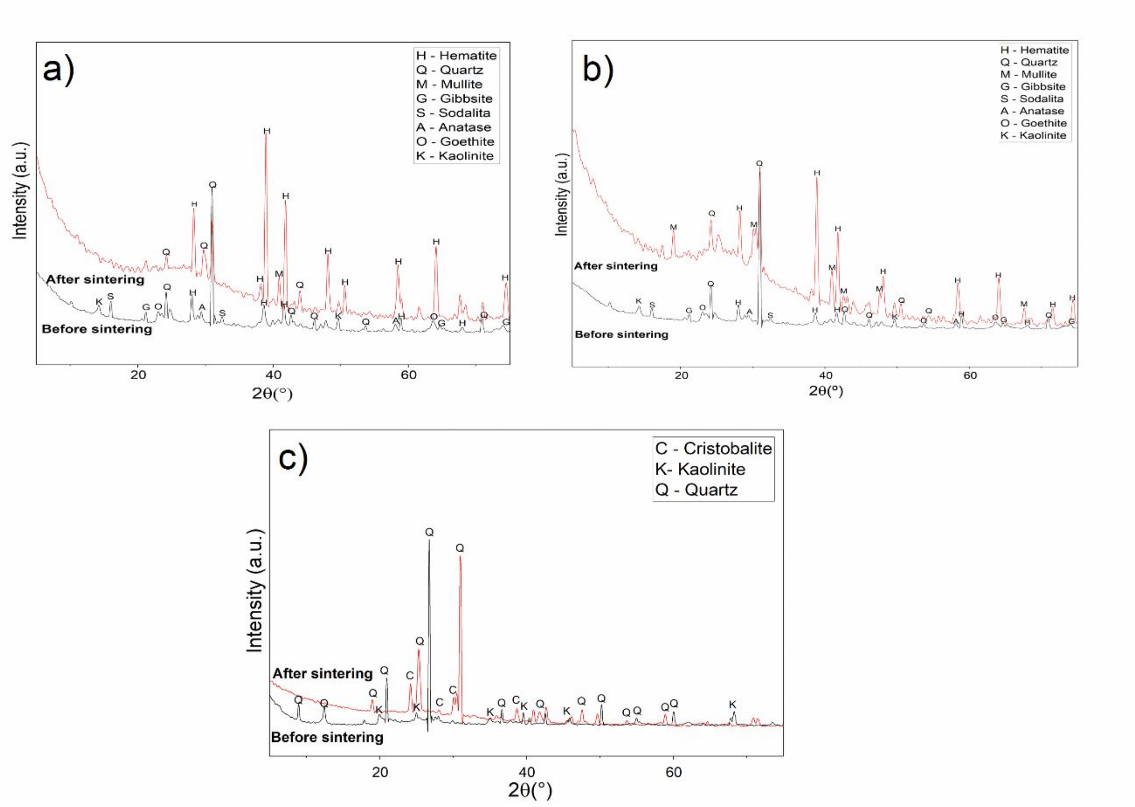

Figure S1 - X-ray diffraction patterns of the samples: (a) BR50, (b) BR30.50, and (c) BR0.
